# Tumor surveillance by circulating microRNAs: a hypothesis

**DOI:** 10.1007/s00018-014-1682-4

**Published:** 2014-07-19

**Authors:** Ivan Igaz, Peter Igaz

**Affiliations:** 1 Department of Gastroenterology, Szent Imre Teaching Hospital, Tétényi str. 12-16, Budapest, 1115 Hungary; 2 2nd Department of Medicine, Faculty of Medicine, Semmelweis University, Szentkirályi str. 46, Budapest, 1088 Hungary

**Keywords:** Nascent tumor, Elimination, Reprogramming, Microvesicle

## Abstract

A growing body of experimental evidence supports the diagnostic relevance of circulating microRNAs in various diseases including cancer. The biological relevance of circulating microRNAs is, however, largely unknown, particularly in healthy individuals. Here, we propose a hypothesis based on the relative abundance of microRNAs with predominant tumor suppressor activity in the blood of healthy individuals. According to our hypothesis, certain sets of circulating microRNAs might function as a tumor surveillance mechanism exerting continuous inhibition on tumor formation. The microRNA-mediated tumor surveillance might complement cancer immune surveillance.

## Introduction

MicroRNAs, the endogenous mediators of RNA interference, have been established as major epigenetic factors in posttranscriptional gene expression regulation. MicroRNAs are encoded by separate genes that are often clustered and undergo a complex maturation process giving rise to 22–25 nucleotide long single-stranded mature microRNA molecules targeting the 3′ non-coding region of messenger RNAs (mRNA) [1]. MicroRNAs either induce target mRNA degradation or inhibition of translation. Approximately, 30–60 % of human protein-coding genes are subjected to microRNA-based regulation [2]. MicroRNAs have pleiotropic actions and are implicated in the regulation of several basic cell biological processes (cell cycle, cell proliferation, apoptosis, etc.), ontogenesis, immune response, hormone secretion, etc. [1]. Alterations in tissue microRNA expression profiles have been associated with several diseases including cancer [2]. MicroRNAs can be classified following the classical oncogene–tumor suppressor dichotomy: overexpressed microRNAs in tumors can be regarded as oncogenes, whereas underexpressed microRNAs are tumor suppressors [3]. The oncogene–tumor suppressor activity of a given microRNA, however, is not absolute, but depends on the cellular context. The same microRNA can be oncogenic in one tissue, but might behave as a tumor suppressor in another. Some microRNAs, however, have either predominant oncogenic or tumor suppressor activity observed in many different tissues (e.g., *hsa*-*miR*-*21*, *hsa*-*miR*-*17/92* families are oncogenic, *hsa*-*miR*-*15/16*, *hsa*-*let*-*7* families are tumor suppressors) [4–7].

Numerous reports underline the relevance of tissue microRNAs both in normal and pathological conditions. Recent data appear to add a further layer of complexity to the biological relevance of microRNAs, as secreted microRNAs have been found in body fluids (blood serum or plasma, urine, semen, saliva, etc.) [8, 9]. MicroRNAs are supposed to enter body fluids via three major mechanisms: (1) passive release from damaged tissue, (2) active secretion in cell-borne membrane vesicles (exosomes, microvesicles and apoptotic bodies), (3) active secretion in macromolecular complexes associated with Argonaute (Ago) proteins (among other still unidentified proteins) and high-density lipoprotein (HDL) particles [10]. The cellular origin of blood-borne serum/plasma microRNAs is not fully explored, but a major source could be represented by blood cells including red and white blood cells, and platelets [11] and the liver [12].

The extracellular microRNAs are surprisingly stable and their analysis is possible even from archived body fluid samples. Circulating blood-borne microRNAs are being investigated as potent minimally invasive biomarkers of several diseases including tumors [13, 14].

Despite their clear relevance as disease markers, the biological relevance of blood-borne microRNAs is largely unknown. Circulating microRNAs can be regarded as hormones that convey epigenetic information and might affect gene expression in cells distant to the cellular microRNA source [15]. Membrane vesicles have been shown to enter other cells and induce changes in gene expression patterns [10]. Tumor cell-secreted microRNAs might be implicated in cell-to-cell communication within the tumor, affect the immune response, and facilitate angiogenesis, tumor invasion and metastatic propagation [16, 17].

The relevance of circulating microRNAs in healthy individuals is, however, entirely unknown. Here, we present a hypothesis on the potential relevance of circulating blood-borne microRNAs.

## Hypothesis: tumor surveillance by microRNAs

The circulating microRNA profiles of healthy individuals have been analyzed in some studies and over 270 different microRNAs have been detected with highly variable expression in different studies on serum or plasma samples [9]. By comparing the relative abundance of these, some microRNAs can be established that are highly overrepresented by normalizing the data of different studies based on the rank orders of the raw expression data [9]. Among the microRNAs that are most abundant, using an arbitrary boundary, i.e., over tenfold overrepresented relative to the 20th most common microRNA in the ranking: *hsa*-*miR*-*451* (106-fold higher expression than that of the 20th microRNA in the ranking) [18], *hsa*-*miR*-*223* (11 to 338-fold) [19, 20], *hsa*-*miR*-*16* (11 to 20-fold) [18, 20] and *hsa*-*let*-*7f* (16-fold) [19] can be highlighted. Two of the three highest ranked microRNAs, *hsa*-*miR*-*335* and *hsa*-*miR*-*377*, in the study by Weber et al. [21] are also worth discussing, but the relative abundance of different plasma microRNAs could not be retrieved from this study.

We have also performed an in silico analysis of the most abundant microRNAs in blood samples of healthy individuals by downloading datasets from Gene Expression Omnibus (www.ncbi.nlm.nih.gov/geo). Data of 61 samples from 5 studies have been retrieved (four of these five studies include data from Asian populations). By rank ordering these microRNAs relative to the 20th most abundant microRNA, *hsa*-*miR*-*451* has emerged among the top-ranked microRNAs in four of the five studies (up to 59-fold overrepresented). *Hsa*-*miR*-*223* and *hsa*-*miR*-*16* are also commonly found in three studies among the 20 most abundant microRNAs. Two further microRNAs worth mentioning: *hsa*-*miR*-*486*-*5p* that is abundant in three independent studies (up to 28.8-fold overrepresented), and *hsa*-*miR*-*923* that is almost 200-fold overrepresented in one study (Table 1).

**Table 1:** The 20 most abundant microRNAs in blood samples of healthy individuals from 5 studies involving 61 samples

GSE25609 (*n* = 20)		GSE53179 (*n* = 5)		GSE39833 (*n* = 10)		GSE41922 (*n* = 22)		GSE50867 (*n* = 4)	
MicroRNA	−Fold^a^	MicroRNA	−Fold^a^	MicroRNA	−Fold^a^	MicroRNA	−Fold^a^	MicroRNA	−Fold^a^
hsa-miR-544	5.1982	**hsa-miR-451a**	59.1044	hsa-miR-923	199.4318	hsa-miR-302a	2.5641	**hsa-miR-451**	1.4702
**hsa-miR-451**	2.2491	**hsa-miR-486-5p**	28.8059	**hsa-miR-451**	36.3352	hsa-miR-145	2.3146	**hsa-miR-16**	1.2892
hsa-miR-302d	2.1451	**hsa-miR-223-3p**	11.1201	hsa-miR-1202	12.5284	hsa-miR-551a	1.5667	**hsa-miR-486-5p**	1.2353
hsa-miR-504	2.0654	hsa-miR-15b-5p	8.8803	hsa-miR-1225-5p	5.1591	hsa-miR-582-5p	1.5053	hsa-miR-19b	1.1863
**hsa-miR-486-5p**	2.0222	hsa-miR-92a-3p	8.4054	hsa-miR-671-5p	4.0057	hsa-miR-181c	1.3988	hsa-miR-92a	1.1433
**hsa-miR-21**	2.0120	**hsa-miR-16-5p**	7.0969	hsa-miR-1299	3.4517	hsa-miR-548c-5p	1.2898	**hsa-miR-223**	1.1348
hsa-miR-550	1.9378	hsa-miR-25-3p	5.7728	hsa-miR-652	1.7898	hsa-miR-338-3p	1.2334	hsa-miR-638	1.1233
hsa-let-7a	1.6534	hsa-let-7a-5p	3.1115	hsa-miR-324-3p	1.6818	hsa-miR-142-5p	1.2326	hsa-miR-22	1.1192
hsa-miR-221	1.6534	hsa-miR-140-3p	2.3809	hsa-miR-144	1.6222	hsa-miR-122	1.1846	hsa-miR-1225-5p	1.1149
hsa-miR-622	1.4578	hsa-miR-107	2.0332	hsa-miR-1268	1.4205	hsa-miR-26b	1.1733	hsa-miR-720	1.0955
**hsa-miR-223**	1.4578	hsa-miR-185-5p	2.0187	hsa-miR-320d	1.2528	hsa-miR-29a	1.1693	**hsa-miR-21**	1.0819
hsa-miR-380	1.3665	hsa-miR-30c-5p	1.9062	**hsa-miR-16**	1.2386	hsa-miR-1537	1.1631	hsa-miR-1207-5p	1.0656
hsa-miR-202	1.2831	hsa-miR-425-5p	1.7028	hsa-miR-142-3p	1.2187	hsa-miR-199a-5p	1.1227	hsa-miR-1915	1.0552
hsa-miR-20a	1.1868	hsa-miR-22-3p	1.5608	hsa-miR-1287	1.2159	hsa-miR-769-5p	1.1190	hsa-miR-320c	1.0464
hsa-miR-122	1.1716	hsa-let-7f-5p	1.4027	hsa-miR-1246	1.1903	hsa-let-7i	1.1175	hsa-miR-1202	1.0456
hsa-let-7g	1.1232	hsa-miR-103a-3p	1.3318	**hsa-miR-223**	1.1449	**hsa-miR-223**	1.1106	hsa-miR-20a	1.0220
hsa-miR-150	1.1087	hsa-miR-19b-3p	1.3038	hsa-miR-188-5p	1.1420	hsa-miR-409-3p	1.1087	hsa-miR-25	1.0161
hsa-miR-551a	1.0221	hsa-let-7b-5p	1.1344	hsa-miR-513a-5p	1.1222	**hsa-miR-21**	1.0196	hsa-miR-15a	1.0159
hsa-miR-25	1.0052	hsa-miR-15a-5p	1.0429	hsa-miR-760	1.0710	hsa-miR-130b	1.0092	hsa-miR-106b	1.0138
hsa-miR-623	1.0000	hsa-miR-320d	1.0000	hsa-miR-720	1.0000	hsa-miR-20a^a^	1.0000	hsa-miR-19a	1.0000

Expression results were downloaded from the Gene Expression Omnibus (GSE25609, GSE53179, GSE39833, GSE41922 and GSE50867). MicroRNAs are ranked by their expression values. The table shows the top 20 microRNA from each study. Data processing and analysis were performed by own programs developed in JAVA program language. MicroRNAs in bold have been found to be relatively abundant in three independent studies. (MicroRNAs denoted with -3p and -5p are transcribed from the same microRNA gene, and represent the two arms of the microRNA precursor)

^a^Values are calculated in relation the 20th highly expressed microRNA (−fold)

Among these microRNAs, several act predominantly as tumor suppressors. *hsa*-*miR*-*16* is one of the most well-known tumor suppressor microRNAs originally described in chronic lymphocytic leukemia [4]. *hsa*-*miR*-*16* is stably expressed in the blood, and it is one of the most widely used reference genes in studies on circulating microRNAs [22]. The *hsa*-*let*-*7* family of microRNAs is regarded as a major group of predominantly tumor suppressor microRNAs that is relevant in lung cancer [5]. Beside *hsa*-*let*-*7f* that has been demonstrated to be relatively abundant compared to other circulating microRNAs [19], other members of this family like *hsa*-*let*-*7a* and *hsa*-*let*-*7g* have also been described as abundant in the circulation [18]. We have also found *let*-*7* family members among the 20 most abundant microRNAs in our in silico analysis (*hsa*-*let*-*7a, hsa*-*let*-*7b, hsa*-*let*-*7f, hsa*-*let*-*7g, hsa*-*let7i*) (Table 1). *hsa*-*miR*-*451*, described to be highly abundant in the sera of Chinese individuals by Chen et al. [18] and also in four of five studies analyzed in silico (Table 1), has been also found to be down-regulated in different tumor tissues (glioma, colorectal cancer, liposarcoma, osteosarcoma) suggesting its tumor suppressor activity [23–28]. The other major blood-borne microRNA that is relatively overrepresented to the others, *hsa*-*miR*-*223* has been implicated in both hematologic and solid tumors, having both oncogenic and tumor suppressor activities depending on the tumor and the cellular context [29]. In gliomas and the Lewis lung cancer cell line, *hsa*-*miR*-*223* behaved as a potent tumor suppressor [30, 31]. The highest ranked microRNA in the study by Tanaka et al. [32], *hsa*-*miR*-*638* shows tumor suppressor activity in gastric cancer [33]. The most abundant microRNA in Weber’s study, *hsa*-*miR*-*335* displays antitumoral activity in a wide variety of different tumors including colorectal [34], gastric [35], ovarian [36] cancers and osteosarcoma [37]. The third most abundant *hsa*-*miR*-*377* by Weber et al. [38] behaves as a tumor suppressor in metastatic prostate cancer cells. (There are no relevant data on the oncogenic relevance of the second most abundant *hsa*-*miR*-*325* in Weber’s study.) Two further microRNAs found to be abundant in our in silico analysis also have tumor suppressor activity: h*sa*-*miR*-*486*-*5p* in breast [39] and lung cancer [40]; *hsa*-*miR*-*923* in chronic lymphocytic leukemia [41].

It must be noted, however, that among the relatively most abundant microRNAs, microRNAs with predominant oncogenic properties can also be found like *hsa*-*miR*-*21* [7] [with ninefold higher expression than the 20th microRNA in the study by Mitchell et al. [19] and also in three of five studies among the top 20 most abundant microRNAs in our in silico analysis (Table 1)]. Among the other relatively abundant microRNAs, several other microRNAs can be found with classical Janus-like activity (tumor suppressor or oncogene depending on the cellular context).

**Fig. 1:**
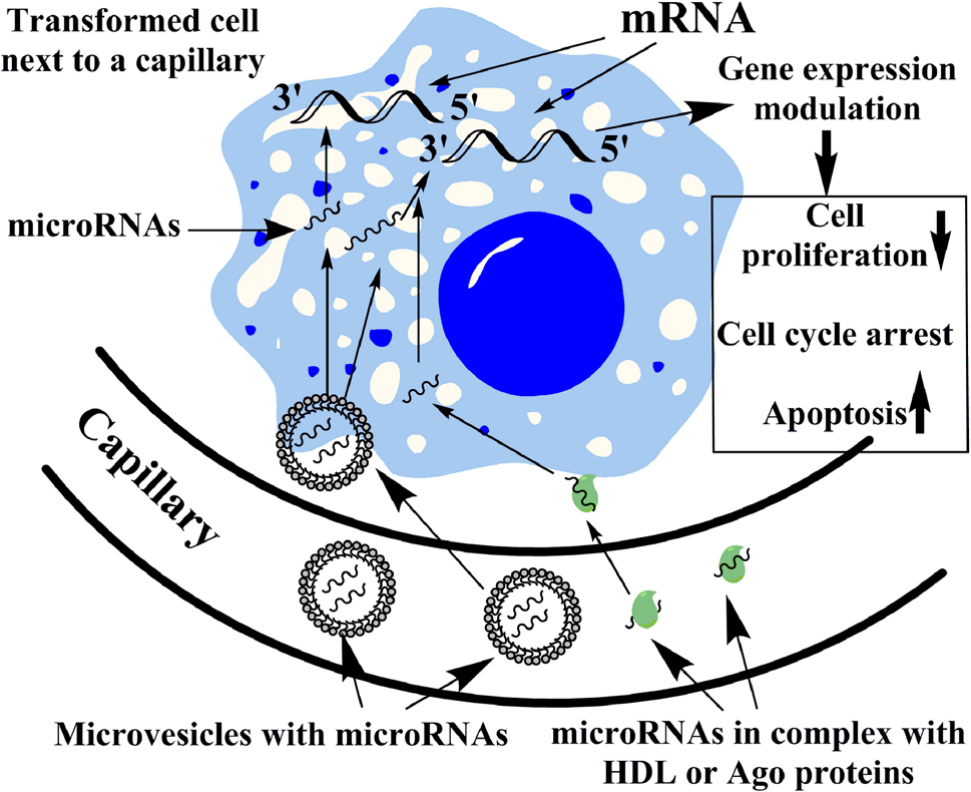
Schematic representation of the potential tumor suppressive action of circulating microRNAs. Circulating microRNAs with predominant tumor suppressor activity included in membrane vesicles (*microvesicles*) or bound in macromolecular complexes with Argonaute (*Ago*) proteins or HDL might enter transformed cells. By targeting mRNAs involved in the regulation of cell cycle, cell proliferation, etc., these microRNAs might result in decreased cell proliferation, cell cycle arrest or apoptosis. This microRNA-mediated tumor surveillance mechanism might represent a continuous inhibition of tumor formation acting in the early phase even preceding or complementing the immune response

The predominant tumor suppressor activity of the most highly overexpressed microRNAs in the blood is intriguing. Is it possible that these microRNAs could inhibit malignant transformation? By entering transformed cells, tumor suppressor microRNAs targeting genes involved in the regulation of cell cycle, cell proliferation, etc., might lead to decreased proliferation, cell cycle arrest or apoptosis induction (Fig. 1). It is hypothesized that potentially malignant cells frequently arise in healthy individuals, and these are destroyed mostly by the immune system according to the paradigm of cancer immune surveillance (cancer immunoediting) [42]. The antitumoral immunity is a very flexible and fast-acting response that eliminates cells potentially harmful to the host [43]. Could blood-borne microRNAs inhibit malignant transformation or proliferation by conveying epigenetic information? If yes, the circulating microRNA pool could present a continuous barrier to tumor formation.

As tissue microRNA dysregulation is considered to be an early event in tumorigenesis [15], alterations in the concentration of microRNAs (i.e., significant reduction in predominantly tumor suppressors) might also be implicated in tumor initiation. Tumor suppressive epigenetic information conveyed by blood-borne microRNAs could reprogram nascent tumor cells by repressing proliferation or inducing apoptosis early in their transformation process and might halt or prevent tumor formation. This hypothesized continuous inhibition of tumor formation might act together with the immune response. As genetic alterations happen much earlier than the immune response that depends on alterations in protein expression, the epigenetic way of tumor inhibition by circulating microRNAs could precede the immune cancer surveillance and complement it. This hypothetical action might add a further layer of host antitumor activity.

Anyway, an extensive research for identifying the mRNA targets of microRNAs isolated from healthy persons and cancerous patients might provide ideas on the tentative tumor suppressive or oncogenic mechanisms of circulating microRNAs, respectively.

Apart from this hypothetical reprogramming of nascent tumor cells in the very early phase of tumorigenesis, blood-borne microRNAs might also influence other, more advanced phases (hallmarks) of tumor formation including angiogenesis, invasion, etc. [44]. Circulating microRNAs might affect angiogenesis [45], and endothelial cells could be the most easily accessible targets cells for circulating microRNAs. It might even be supposed that the antitumoral immunity could be affected by blood-borne microRNAs, as well, and thereby blood-borne microRNAs might not only complement, but also might interact with immune cancer surveillance.

An interesting similar hypothesis on the relevance of circulating microRNAs in tumors has been put forward by Chen et al. [12], who observed that in the blood of cancer patients microRNAs with predominantly tumor suppressor activity are more abundant than oncogenic microRNAs. The relative abundance of tumor suppressor microRNAs can be viewed as a form of anti-cancer defense mechanism in advanced cancer patients [12]. Chen et al. proposed that the anti-cancer action of these microRNAs might be predominantly mediated via enhancing the antitumor immune response. Our hypothesis, on the other hand, concerns tumor suppressor microRNAs in healthy individuals, whereby these could constitute a form of antitumor surveillance reprogramming nascent tumor cells and inhibiting tumor formation in its early phase.

## Counter arguments and problems

A major counter argument against this hypothesis could be the cellular context-dependent activity of microRNAs. Even microRNAs with predominant tumor suppressor activity could have oncogenic relevance in certain tissues. Our tenfold boundary (tenfold overrepresented to the 20th microRNA in ranking) for discussing the most relevant circulating microRNAs is certainly arbitrary, and among the microRNAs below this threshold, several others can be found having both oncogenic and tumor suppressor activity or even predominantly oncogenic like *hsa*-*miR*-*21*. The relative abundance of microRNAs with predominantly tumor suppressor activity in the over tenfold overrepresented group is, however, noteworthy.

Another major problem is related to the very low quantity of circulating microRNAs. Even the concentration of the most overrepresented circulating microRNAs is very low, and it is unknown whether microRNAs in such low concentration are potent enough to result in significant gene expression alterations in cells internalizing them. There are in vitro data supporting the gene expression modulating capacity of both membrane vesicle and lipoprotein-associated microRNAs [10, 46], but there are no findings whatsoever showing that microRNAs in the circulation can indeed mediate gene expression information to tissues.

A further issue is related to the affected cell populations. Apart from the endothelial and blood cells getting into direct contact with circulating microRNAs, what other cells could be affected and how? Most probably, microRNAs can traverse the capillary endothelium, as there are data that even exogenous microRNAs might pass the gastrointestinal epithelium and enter the circulation [47, 48]. The gastrointestinal transfer of microRNAs, however, is not universally accepted, and other research findings indicate that this process is not efficient [49]. Exosomes have been shown to traverse the blood–brain barrier [50]. It is not known, however, whether protein or HDL-bound microRNAs can also do the same. The molecular mechanisms responsible for these phenomena have not been defined, yet. Nevertheless, the different forms of storage of microRNAs (unbound, extracellular vesicle bound or protein protected) might result in different half-lives of microRNAs. Moreover, the selectivity of the storage forms of different microRNAs cannot be excluded.

There are certainly interindividual and ethnic variations in microRNA profiles of healthy individuals as exemplified by the findings of different research groups (Table 1). Habits, e.g., smoking might significantly influence the microRNA profile, as well [51]. It is, therefore, difficult to establish a general “normal” profile. Methodological problems might also account for variations, since difficulties of standardized microRNA isolation, platform differences (microarray, real-time polymerase chain reaction-based techniques, next generation sequencing), and the choice for housekeeping might also affect the results.

It will be rather difficult to test this hypothesis experimentally. In vitro experiments could be conceived to test whether microRNAs could halt tumorigenesis or induce apoptosis in nascent tumor cells generated by, e.g., viral or chemical transformation induction processes in cell cultures. As our hypothesis relates to the inhibition of nascent tumor formation in vivo, animal tumor models should be examined. microRNAs with predominant tumor suppressor action could be administered to mouse tumor models (e.g., spontaneous or chemical induced tumors in immune-deficient mice) and the frequency of tumor formation could be tested. Several problems need to be addressed in these studies, e.g., form of administration, the concentration of the microRNA administered, potential off-target microRNA actions, etc. Alternatively, knockout or transgenic mouse models of selected microRNAs abundant in the circulation could be examined for spontaneous or induced tumor frequency. In the available knockout mouse models for *hsa*-*miR*-*223* and *hsa*-*miR*-*451*, however, no increase in spontaneous tumor frequency has been reported [52, 53]. Longer observation periods and tumor induction might be envisaged in these models. The tumor surveillance by microRNAs, however, would not be a phenomenon involving only a single microRNA, rather microRNA sets, and therefore combinations of different microRNAs with tumor suppressor activity should be tested.

## Conclusions

Here, we have presented a hypothesis on the potential relevance of circulating, blood-borne microRNAs in healthy individuals. Our hypothesis is based on the relative abundance of microRNAs with predominant tumor suppressor activity among the most highly overrepresented microRNAs in serum or plasma. Their potential activity as tumor suppressors might be intriguing. It must be noted, however, that we have highlighted only one potential activity of circulating microRNAs in healthy individuals. Circulating microRNAs might affect other major homeostatic mechanisms, i.e., immune response, hematopoiesis, etc. Further studies will be necessary to gain deeper insight into this fascinating field and to be able to explore the biological relevance of circulating microRNAs.
